# Two fork protection complexes at the replication fork play distinct roles in fork progression and stress response

**DOI:** 10.21203/rs.3.rs-9888792/v1

**Published:** 2026-06-03

**Authors:** Sameera Vipat, Rohan Harolikar, Naga Raviteja Chavata, Karina Šapovalovaitė, Syed Shahid Musvi, Arthur Morgunov, Sigvard Vällo, Tatiana N. Moiseeva

**Affiliations:** 1 Department of Chemistry and Biotechnology, Tallinn University of Technology, Tallinn, 12618, ESTONIA; 2 Department of Pharmacology and Chemical Biology, University of Pittsburgh, 15213, Pittsburgh, PA; 3 Vilnius University, Vilnius, 01513, LITHUANIA; 4 UPMC Hillman Cancer Center, 15213, Pittsburgh, PA

## Abstract

TIMELESS, together with TIPIN and CLASPIN, forms the Fork Protection Complex (FPC), an essential regulator of DNA replication that possesses multiple functions in genome stability including the regulation of fork progression and replication checkpoint signaling. Structural studies place TIMELESS at the leading edge of the CMG helicase, which is inconsistent with FPC functions at the lagging strand and on single-stranded DNA. Our observation that FPC chromatin loading during replication initiation started in G1 phase cells, but was also enhanced by DNA synthesis, led us to propose a model of a step-wise loading of the FPC with two TIMELESS molecules per replication fork. Split-TurboID proximity labelling supported this model, placing the second FPC in proximity to the lagging strand. Using an auxin-inducible degron, we show that TIMELESS depletion compromised chromatin loading of TIPIN and CLASPIN, but the TIMELESS mutant unable to bind MCM still supported CLASPIN and TIPIN chromatin loading. This mutant was proficient in replication checkpoint activation, but failed to regulate fork speed under both unperturbed and oxidative-stress conditions. We propose that two distinct FPC instances at each replication fork: one at the leading edge, regulating fork progression, and one at the lagging strand mediating checkpoint signaling, - together execute the essential functions of TIMELESS.

## Introduction

Timely and accurate DNA replication is essential for cell proliferation and genome stability. TIMELESS is a part of the Fork Protection Complex (FPC, TIMELESS-TIPIN-CLASPIN) that has several important functions during unperturbed replication as well as in replication stress response (1–4). TIMELESS performs most of its functions bound to its obligate binding partner TIPIN, and a depletion of one partner leads to a downregulation of the other(5). The best described role of TIMELESS at the replication fork is the regulation of replication fork speed – both under normal conditions (6) and after oxidative stress (7). TIMELESS-TIPIN have been shown to coordinate the activities of the MCM helicase and the polymerases (6, 8–10) by stimulating DNA polymerase activity and regulating MCM helicase activity at the same time(8, 11), ensuring the maintenance of normal replication fork speed. TIMELESS-PARP1 interaction was shown to mediate proper lagging strand synthesis and response to stalled forks (6, 12). Additionally, FPC interaction with RPA has been reported to be an integral part of the regulation of replication checkpoint signaling (4). More recently, a new function of TIMELESS has been uncovered: TIMELESS/CLASPIN complex blocks PAF15 binding to PCNA on the leading strand, preventing misregulation of PCNA unloading (13).

Taking into account this diverse array of functions, it is important to establish the position of TIMELESS at the replication fork. Structural studies have proven beyond any doubt that TIMELESS is positioned ahead of the advancing CMG helicase (14–16), at the leading edge of the replisome. However, direct interaction between TIPIN and RPA/ssDNA (4) and the role of TIMELESS in the regulation of lagging strand synthesis (12), suggest a position for the FPC at the ssDNA between Okazaki fragments. Additionally, biochemical evidence suggested that TIMELESS interacts with the polymerases at the fork and stimulates their activities(8, 11), and its newly discovered role in PCNA regulation (13) is also difficult to reconcile with its position at the leading edge of the replication fork. In this study we propose a model in which two FPCs located at different positions at the replication fork are responsible for distinct functions of the FPC.

Replication initiation follows a conserved program in eukaryotes (17, 18). Origins are licensed in G1 by loading inactive MCM double hexamers, after which CDK- and DDK-dependent steps drive CMG helicase assembly through recruitment of CDC45 and GINS. Following primer synthesis by primase and initial DNA synthesis by polymerase alpha, PCNA loading by RFC (19) triggers polymerase delta recruitment(20), before a polymerase switch establishes leading-strand synthesis by polymerase epsilon (21). Two diverging replication forks are then established. When the FPC is recruited to the replication complex is still poorly understood. One study indicated that TIMELESS is loaded to origins with the same dynamics as CDC45(22), while another study reported that TIMELESS associates with MCM even before the beginning of S phase (23), which may indicate that it is loaded to origins along with MCM in G1. The latter model is not consistent with the TIMELESS location at the leading edge of the fork which would be concealed in the MCM double hexamers according to structural data (24, 25). What role, if any, TIMELESS-TIPIN play in replication initiation is also not clear. Some studies showed excessive origin firing in TIMELESS-depleted cells (5) and others reported delayed S phase entry and a reduction in the S phase cell population (23) upon TIMELESS depletion.

Here, using cell synchronizations and PLA, we show that TIMELESS recruitment to MCM begins in G1 phase of the cell cycle, before the start of DNA synthesis, but it is supported by the activities of the S-phase kinases and by the DNA synthesis. This prompted us to hypothesize that TIMELESS is recruited stepwise, with different instances of FPC taking different positions at the replication fork.

Using a split-TurboID-based proximity labelling system (26) to study the proteins in proximity to TIMELESS at the fork, we show that the set of proteins biotinylated by the used split-TurboID pairs supported the model in which there is an additional FPC at ssDNA on the lagging strand. Further, using a mutant of TIMELESS unable to interact with MCM but retaining its interactions with TIPIN and CLASPIN, we show that TIMELESS-MCM interaction is essential for the role of TIMELESS in regulation of replication fork speed – under normal conditions and during oxidative stress. Our data show that while TIMELESS is essential for chromatin loading of TIPIN and CLASPIN, TIMELESS interaction with MCM was not needed for chromatin loading of the FPC to the second position, likely at the lagging strand, or for its role in replication checkpoint signaling.

Our data support a model where two instances of the fork protection complex at the replication fork play distinct roles in regulating replication fork progression and replication stress response.

## Results

### TIMELESS chromatin loading during origin firing requires CMG assembly and the initiation of DNA synthesis

In order to better understand the recruitment of TIMELESS, TIPIN and CLASPIN to replication complexes during S-phase entry, we studied the chromatin loading of TIMELESS, TIPIN and CLASPIN relative to the loading of other replication proteins using cell synchronization.

**Figure 1.**
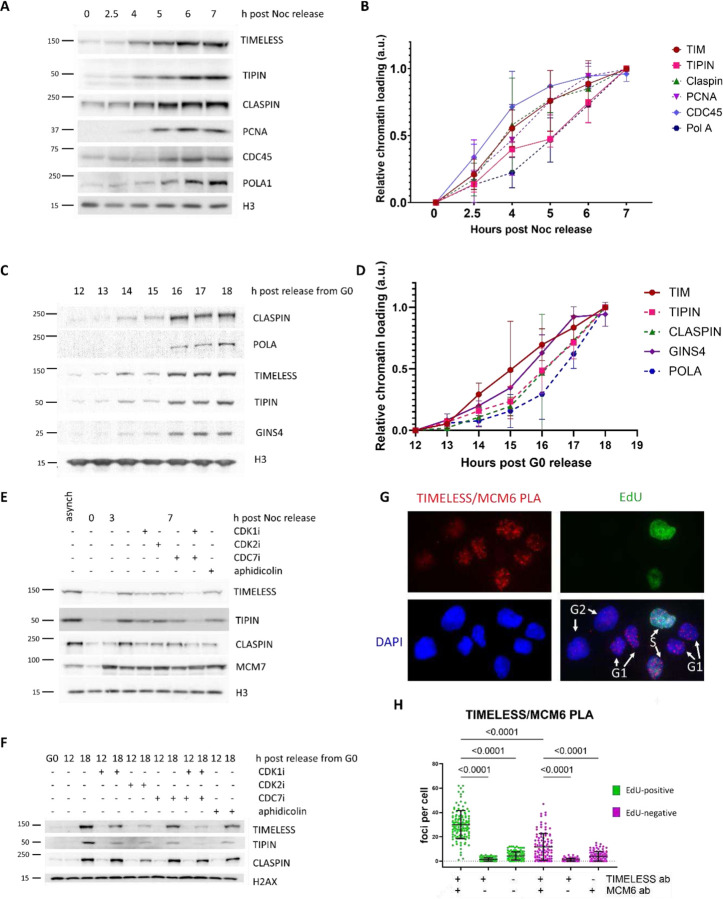
Timing and regulation of FPC chromatin loading during replication initiation. **A-D.** U2OS and RPE1-hTERT cells were synchronized as indicated on **Fig. S1A, C.** Samples were collected at the indicated timepoints post nocodazole release. Western blot analysis of the chromatin fraction from the cells collected at the indicated timepoints is shown (**A, C)**. Quantifications of protein loading from A and C are shown -mean + SD from 3 independent experiments (**B, D**). **E-F.** 3h post nocodazole release (for U2OS (**E**)) or 10h post release from G0 (RPE1-hTERT (**F**)), the indicated inhibitors were added. Samples were collected at the indicated timepoints post nocodazole release. Western blot analysis of the nuclease-insoluble chromatin fraction from the cells is shown (**E-F**). G-H. U2OS cells were pulsed with EdU for 30 min, followed by CSK extraction, fixation, and PLA staining. The experiment was repeated 3 times, representative microscopy images (**G**) and a quantification of a representative repeat (~100 cells per condition)(**H**) are shown. One-way ANOVA was used for statistical analyses.

We synchronized U2OS using the double thymidine-nocodazole block (**Fig. S1A**), collected cells at the indicated timepoints after their release from nocodazole (from G2 through G1 and S-phase entry), and analyzed the chromatin fraction by western blot (Fig. 1A–B, **S1B).** TIMELESS and CLASPIN loaded concurrently with CDC45 (a marker of CMG assembly), but notably before DNA polymerase alpha, indicating that DNA synthesis was not required to initiate FPC chromatin loading during S-phase entry. Similar results were obtained with RPE1-hTERT cells synchronized using serum starvation (**Fig. S1C**) and contact inhibition, confirming that TIMELESS chromatin loading was initiated before DNA polymerase alpha recruitment (Fig. 1 C–D**, S1D**).

Further, to identify the steps of replication initiation required for FPC chromatin loading, we treated synchronized U2OS or RPE1-hTERT cells with the inhibitors of the kinases CDK1, CDK2 or CDC7, known to be involved in replication initiation (27, 28), or the B-family DNA polymerase inhibitor aphidicolin. Cell cycle analysis confirmed that the inhibitors we used affected S phase entry in both cell lines (**Fig. S1E-G**). FPC chromatin loading was affected by CDK1, CDK2 and CDC7 inhibitors, and the combination of CDK1i and CDC7i almost completely suppressed FPC chromatin loading (Fig. 1E–F**, S1H-I**) in both cell lines. Since these kinase activities are known to regulate CMG assembly (27), we concluded that CMG assembly was likely required for FPC chromatin loading. Interestingly, aphidicolin also decreased FPC chromatin loading, indicating that DNA synthesis was needed for the stable association of FPC with chromatin (Fig. 1E–F**, S1H-I**). Given that TIMELESS and CLASPIN associated with chromatin before POLA was recruited (Fig. 1A, B), DNA synthesis could not be the step that initiated FPC loading.

In order to obtain a clearer picture of TIMELESS association with MCM during the cell cycle, we used a proximity ligation assay with antibodies against TIMELESS and MCM6 (the only MCM antibody shown to retain interaction with MCM in an active replication fork (29)), after pre-extracting cells to remove soluble proteins. Our data (Fig. 1G–H) showed the presence of specific MCM-TIMELESS PLA foci in EdU-negative G1 cells. Larger EdU-negative nuclei, presumably G2, contained a background level of PLA foci, in agreement with MCM having been unloaded from chromatin in those cells. EdU-positive cells contained a significantly higher level of TIMELESS/MCM6 PLA foci than EdU-negative cells. These data were in agreement with the western blot data, suggesting the existence of two steps of replication initiation that regulate FPC association with chromatin – one in G1 and one during the initiation of DNA synthesis.

### TIMELESS depletion leads to a defect in chromatin loading of CLASPIN and TIPIN

In order to study the role of TIMELESS in replication initiation in human cells, we used a mini-auxin-inducible degron (mAID2) system (30) to achieve rapid and near-complete depletion of TIMELESS. In this system, the C-terminus of the target protein TIMELESS is tagged with a mAID-mCherry tag using CRISPR genome modification. The modified F-box protein osTIR1 F74G allows for a faster degradation (3–4 hours).

**Figure 2.**
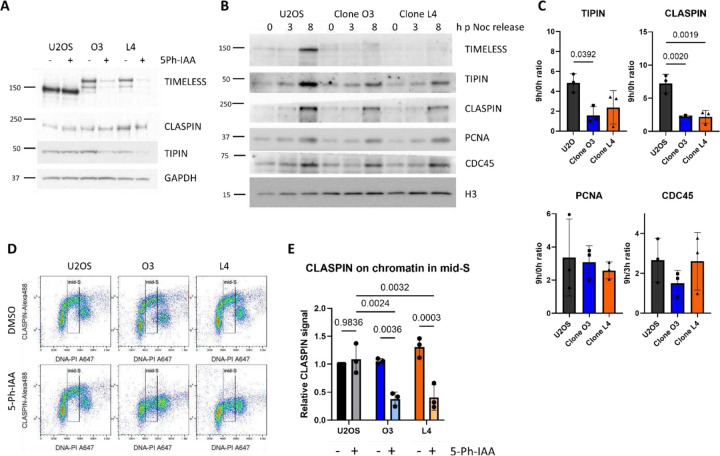
Effect of TIMELESS depletion on chromatin loading of replication proteins. **A.** U2OS, mAID2 clones Clone O3, Clone L4 were treated with auxin 5-pH-IAA for 24h. Western blots of total cell lysates are shown (**A**). **B-C.** U2OS, mAID2 clones O3 and L4 were synchronized using the double thymidine/nocodazole block, 5-Ph-IAA was added at the time of the release from nocodazole. Western blot analysis of the CSK chromatin fraction from the cells collected at the indicated timepoints is shown (**B**). Quantifications of protein loading (mean + SD from 3 independent experiments) are shown (**C). D-E.** U2OS, mAID2 clones O3 and L4 were treated for 16h with 5-Ph-IAA, as indicated. Cells were extracted with CSK buffer and stained with antibodies against CLASPIN and a DNA dye. Representative flow cytometry experiment (**D**) and quantification (mean + SD from 3 independent experiments) (**E**) are shown.

Two U2OS-based clones - Clone O3 and Clone L4 were selected, based on their ability to deplete TIMELESS to near-complete levels, and, as expected, its partner TIPIN was concurrently downregulated after a 24-h TIMELESS depletion (Fig. 2A). The levels of CLASPIN, the other FPC component, were not affected by TIMELESS depletion(Fig. 2A).

While TIMELESS depletion did not significantly affect the percent of EdU-positive cells within 24h of treatment (**Fig. S2A**), it significantly suppressed the cell growth within 72h (**Fig. S2B**), in agreement with the essential role of TIMELESS in cell proliferation.

Next, in order to investigate the effect of TIMELESS depletion on S phase entry, we depleted TIMELESS in cells that were synchronized in G2/M using a double thymidine-nocodazole block and then allowed to enter the S phase (**Fig. S1A**). We added 5-Ph-IAA to the cells at the time of release from nocodazole. Cells were collected at the indicated timepoints following the release from nocodazole, marking G2/M, G1, and S-phase entry. mAID tagging had no effect on the cell cycle progression after synchronization without TIMELESS depletion (**Fig. S2C**).

Quantifications of S phase cells (**Fig. S2D**) showed no delay in S-phase entry in TIMELESS-depleted clones O3 and L4. However, DNA synthesis as measured by the level of EdU incorporation was lower in both TIMELESS-depleted clones compared to U2OS cells (**Fig. S2E**), which may be at least partly due to the previously documented (6) slower fork speed upon TIMELESS depletion. In order to check whether this effect is more pronounced in early S-phase cells that we studied by synchronization, we performed EdU incorporation analysis on asynchronous TIMELESS-depleted cells separating S-phase cells into early-S, mid-S, and late-S (**Fig. S2F-G**). Early S-phase cells were not more affected by TIMELESS depletion than other fractions of the S-phase. Indeed, the effect on EdU incorporation in synchronized cells (**Fig. S2G**) was much more pronounced (~50%) than the effect on any particular fraction of S-phase in the asynchronous cells (~25%). This may indicate that in TIMELESS-depleted samples there were more very early S-phase cells known to have slower EdU incorporation (31), suggesting a slower S-phase entry in the absence of TIMELESS.

Aiming to determine whether TIMELESS depletion affected chromatin loading of any replisome components, we synchronized mAID clones and U2OS cells using the double thymidine-nocodazole block, depleted TIMELESS as described above, and analyzed the presence of selected replication proteins in the chromatin fraction using western blot at indicated timepoints. At 3–8h after 5-Ph-IAA addition, TIMELESS was completely depleted in the soluble fraction, while TIPIN was barely affected (**Fig. S2H**). Interestingly, TIPIN chromatin loading was significantly reduced in TIMELESS-depleted cells (Fig. 2B–C). We also observed a decrease in the chromatin recruitment of CLASPIN (Fig. 2B–C) during S-phase entry. CDC45 and PCNA chromatin loading during S-phase entry was not affected by TIMELESS depletion, indicating that there was no noticeable delay in replication initiation. Our data showed that TIMELESS depletion affects the chromatin loading of CLASPIN and TIPIN during S-phase entry. We also confirmed a strong effect of TIMELESS depletion on CLASPIN chromatin loading using flow cytometry analysis of chromatin-extracted cells stained with an antibody against CLASPIN (Fig. 2D–E)

To confirm our findings using an alternative approach, we used an ATR inhibitor AZD6738 (ATRi) which is known to rapidly induce massive origin firing resulting in a strong recruitment of replication proteins to chromatin, MCM4 phosphorylation and an increase in EdU incorporation by replicating cells (32). We depleted TIMELESS in mAID clones and added DMSO or 5 μM ATRi (AZD6738) for the last 1 h to induce origin firing. We observed significant reductions in CLASPIN and TIPIN recruitment to nuclease-insoluble chromatin in response to ATRi in TIMELESS-depleted clones (**Fig. S2I-J**), while the recruitment of CDC45 and GINS remained unaffected. These data demonstrate that TIMELESS was required for the recruitment of CLASPIN and TIPIN to chromatin during the initiation of DNA replication for the proper assembly of the FPC at the replication fork.

### Proximity labelling enabled by TIMELESS interaction with core replication initiation proteins indicates the presence of more than one molecule of TIMELESS per fork

**Figure 3.**
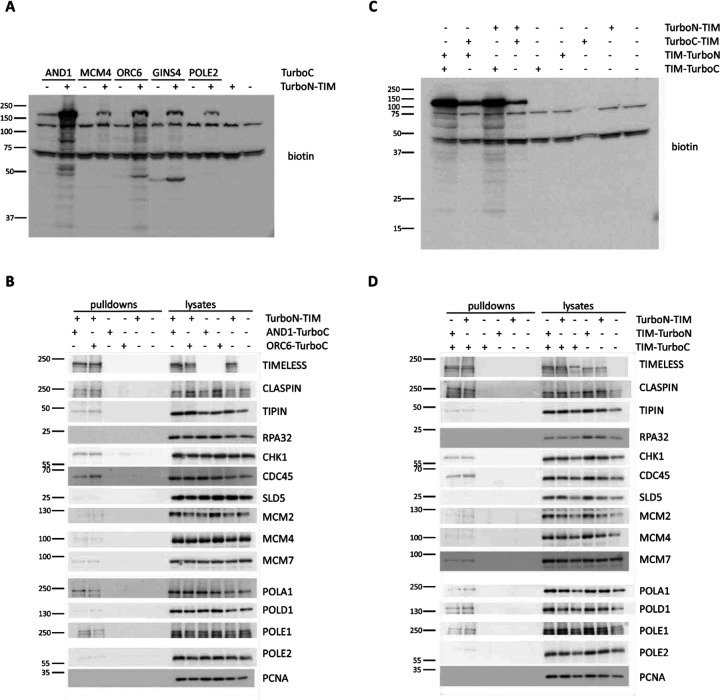
Split-TurboID approach uncovers TIMELESS-proximal proteins at the replication fork. **A-D.** HEK293T cells were transfected with the plasmids expressing indicated TurboN- or TurboC-tagged proteins. 48h after transfection cells were treated with 50 μM biotin for 1 hour and lysed. (**A, C**) Cell lysates were analyzed by western blot using antibodies against biotin (**A, C**). Western blot analyses of the streptavidin pulldowns and the lysates’ samples are shown (**B, D**).

In order to shed some light on the position of TIMELESS in the replisome and possible mechanisms of recruitment, we decided to look into the proteins located in proximity to TIMELESS at the replication fork. We therefore employed a split-Turbo ID-based proximity labelling strategy (26), allowing biotinylation of nearby proteins only when two bait proteins tagged with two complementary parts of TurboID biotin ligase (TurboN and TurboC) come together to form a functional enzyme. To identify suitable replication proteins to be combined with TIMELESS for this approach, we tagged several replication proteins (MCM4, AND-1, ORC6, POLE2) with TurboC and combined each of them with TurboN-TIMELESS. TIMELESS-ORC6 and TIMELESS-AND-1 combinations yielded the best overall biotinylation signal (Fig. 3A**, S3A**).

AND-1 is a trimer positioned on the other side of the CMG helicase with respect to the N-terminus of TIMELESS, making it an unlikely successful combination. ORC6 is a subunit of the Origin Recognition Complex, which does not travel with the replication fork. However, ORC6 has been shown to also participate in mismatch repair behind the fork (33), and if this ORC6 molecule forms a successful split-TurboID pair with TIMELESS, TIMELESS must be positioned close to one of the daughter strands.

To clarify the locations of contacts for the two successful combinations, we performed a streptavidin pulldown, and looked for known components of the replication fork among the proteins biotinylated by TIMELESS-AND-1 and TIMELESS-ORC6 pairs. Apart from the FPC components, the proteins showing the strongest biotinylation were CDC45 and CHK1, followed by DNA polymerases alpha and epsilon (Fig. 3B). Interestingly, we did not observe any noticeable differences in biotinylation levels for the selected proteins between the two combinations used in the experiment. Biotinylation of DNA polymerase epsilon on the leading strand and CHK1 (likely located close to ssDNA on the lagging strand) was difficult to reconcile with the canonical position of TIMELESS at the leading edge of the replication fork on the parental DNA.

While this canonical position of TIMELESS has been repeatedly demonstrated beyond any doubt (14–16), we decided to check if there could possibly be more than one TIMELESS molecule per replication fork.

To this end, we used split-TurboID combinations in which two TurboID parts were fused to two molecules of TIMELESS, both N-terminal and C-terminal tagging were tested (Fig. 3C**, S3B**). Interestingly, TIMELESS-TurboC produced strong biotinylation in combination with either TurboN-TIMELESS or TIMELESS-TurboN, indicating two molecules of TIMELESS being positioned in proximity to each other. TurboC-TIMELESS did not produce strong biotinylation in any combinations, probably due to TurboC being a larger tag that could be disruptive at the N-terminus of TIMELESS.

Analysis of proteins biotinylated by TIMELESS-TIMELESS combinations confirmed the presence of the same set of proteins that we saw biotinylated by TIMELESS-AND-1 and TIMELESS-ORC6 combinations (Fig. 3D). Interestingly, in addition to these proteins, the TIMELESS-TIMELESS combinations also biotinylated DNA polymerase delta, placing at least one molecule of TIMELESS in proximity to the lagging strand. Given that TIPIN was shown to interact with RPA and ssDNA (4), we propose that in addition to the leading edge FPCs, there could be secondary TIMELESS/TIPIN complexes at the ssDNA gaps on the lagging strand.

### Disrupting the interaction between MCM and TIMELESS does not prevent FPC chromatin association, but does disrupt its stable association with the CMG helicase

**Figure 4.**
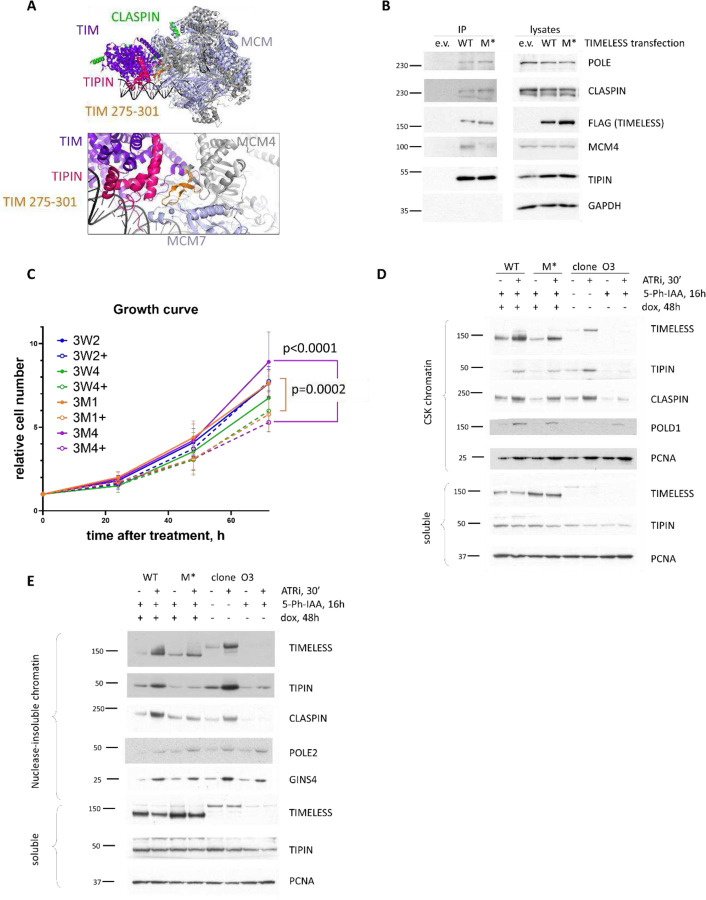
TIMELESS-MCM interaction is essential for cell growth, but not for the chromatin loading of the FPC. **A.** The position of the deletion in TIMELESS resulting in the disruption of TIMELESS-MCM interaction is shown on the experimental structure (PDB:7PFO (14)). **B.** Constructs expressing FLAG-tagged WT TIMELESS or TIMELESS-M* were transfected in 293T cells. 48h after transfection, TIMELESS was immunoprecipitated and eluted from the M2 beads using FLAG peptide. Western blot analyses of the indicated proteins are shown. **C.** Equal numbers mAID2 clone O3, and clones based on O3 expressing WT TIMELESS (W2 and W4) or TIMELESS M* (M1 and M4), were seeded on 60 mm dishes and treated with DMSO or dox/5-Ph-IAA for 72 h. Cell numbers were counted every 24 h and growth curves were plotted (mean +/− SD from n = 3 independent experiments). **D-E.** mAID2 clone O3, and clones based on O3 expressing WT TIMELESS (W2) or TIMELESS M* (M1), were treated with 10ng/ml doxycycline for 48h and 5Ph-IAA for 16h. ATRi or DMSO was added for 30 min. Western blot analyses of CSK chromatin (D) or nuclease-insoluble chromatin (E), as well as soluble fractions are shown.

In our proposed model at least two TIMELESS/TIPIN complexes are present at the replication fork, however, only one molecule of TIMELESS interacts with MCM. To clarify whether TIMELESS-MCM interaction is needed for the roles of TIMELESS in DNA replication, we generated a mutant of TIMELESS unable to interact with MCM - TIMELESS-M*. We designed it based on the data from (8), showing that TIMELESS amino acids 1–663 were sufficient for MCM interaction, but neither of the two broader regions present on either side of region 1–663 (1–307 or 308–1208) was sufficient for MCM interaction(8). We deleted the amino acids 275–301 forming a loop next to the MCM hexamer (Fig. 4A). We also took into consideration structural data (14–16), and used Phyre2 (34) and AlphaFold (35) to ensure our deletion would cause minimal changes to the 3D structure of TIMELESS, aiming to preserve its interactions with its other partners. This mutant was predicted to have weaker interactions with MCM using Alphafold3 (**Fig. S4A**), and the disruption of TIMELESS-M* interaction with MCM was further confirmed using co-immunoprecipitation, while its interactions with TIPIN, CLASPIN and POLE were not affected (Fig. 4B).

To further test the effect of disrupting the interaction between TIMELESS and MCM on DNA replication, we expressed WT TIMELESS or TIMELESS-M* under the doxycycline-inducible promoter in the mAID2 clone O3 able to deplete endogenous TIMELESS. We initially selected two clones for each variant of TIMELESS – W2 and W4 expressed WT TIMELESS while M1 and M4 expressed TIMELESS-M*. Just as TIMELESS-depleted cells (**Fig. S2B**), TIMELESS-M*-expressing cells showed a significant decrease in proliferation (Fig. 4C).

We chose clones W2 (WT TIMELESS) and M1 (M* TIMELESS) for further analysis because they showed similar proliferation rates without treatments.

To check whether the interaction between MCM and TIMELESS is essential for FPC chromatin loading, we first used ATRi-induced origin firing to observe the loading of replisome components onto chromatin and increase in EdU incorporation. Disruption of MCM-TIMELESS interaction strongly decreased FPC chromatin loading in response to ATRi (**Fig. S4B**). Recruitment of CMG components CDC45 and GINS4 to chromatin, and an increase in EdU incorporation in response to ATRi were not affected by the disruption of TIMELESS-MCM interaction (**Fig. S4B-C**), in agreement with TIMELESS not being required for origin firing.

Throughout this study we have been using two different approaches for chromatin fractionation: CSK buffer extraction uses Triton X-100 to extract soluble proteins (used in synchronization experiments), and nuclease-insoluble chromatin (NIC), analyzed in experiments using ATRi-induced origin firing, uses nuclease for additional extraction. The key difference is that lagging-strand factors loosely associated with the CMG — such as PCNA and DNA polymerase delta — are retained on CSK chromatin (Fig. 1A, C, 4D) but lost from NIC (32). We therefore decided to check whether TIMELESS-M* can be recruited to these two types of chromatin fractions during the initiation of DNA replication. ATR inhibitor-induced replication origin firing resulted in strong recruitment of TIMELESS-M* to CSK chromatin, along with other FPC components, as well as POLD and PCNA (Fig. 4D). However, recruitment of TIPIN and CLASPIN to NIC was strongly decreased in M*-expressing cells compared to WT TIMELESS, while recruitment of GINS4 and POLE2 was unaffected by TIMELESS mutation (Fig. 4E). These data indicate that M* TIMELESS is capable of recruiting TIPIN and CLASPIN to the chromatin during replication initiation, but in the absence of TIMELESS interaction with MCM, FPC complex is recruited by a different interaction (likely TIPIN-RPA), and not tightly associated with the CMG helicase, supporting the model for two FPCs at the replication fork.

### The effect of disrupting TIMELESS/MCM interaction on the functions of TIMELESS at the replication fork.

**Figure 5.**
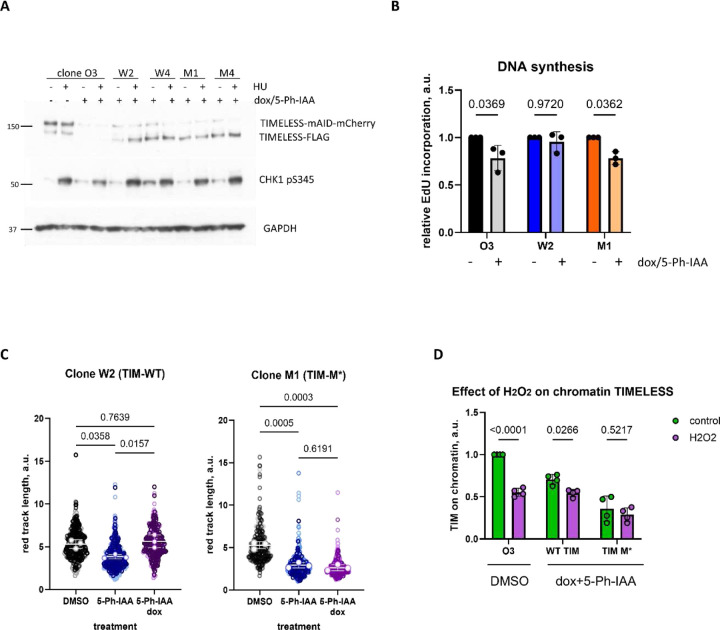
The effect of disrupting TIMELESS/MCM interaction on the functions of TIMELESS at the replication fork. **(A)** mAID2 clone O3, and clones based on O3 expressing WT TIMELESS (W2 and W4) or TIMELESS M* (M1 and M4), were treated with 10 ng/ml doxycycline and 5Ph-IAA for 16h. 2mM HU was added for 1h as indicated. Western blot analyses on total cell lysates are shown. (B) After a 30 min EdU pulse, quantification of EdU incorporation by FACS is shown, based on three independent experimental repeats **. (C)** Cells were pulsed with 20 μM CldU for 10 min followed by 200 μM IdU for 20 min, and lysed followed by DNA fiber analysis. Quantification of red track length (CldU) based on three independent experiments, is shown**. (D)** Clones based on O3 expressing WT TIMELESS (W2) or TIMELESS M* (M1), were treated with 10ng/ml doxycycline for 48h and 5Ph-IAA for 16h, mAID2 clone O3 was treated with DMSO for 16h. 50μM H_2_O_2_ was added to the cells for 30 min before harvest. Quantification of the relative level of TIMELESS on chromatin in the mid-S-phase is shown (mean +/− SD from n = 4 independent experiments). One-way ANOVA (**C**) or two-way ANOVA (**B, D**) were used for statistical analyses.

In order to have a better understanding of which functions of TIMELESS are associated with the leading edge vs. lagging strand instance of the FPC, we first looked at the ability of TIMELESS-M* to support the activation of the replication checkpoint signaling. While TIMELESS depletion in clone O3 decreased CHK1 phosphorylation after replication stress induction by hydroxyurea (HU) treatment, expression of either WT or M* TIMELESS was sufficient to support checkpoint signaling activation (Fig. 5A). This confirmed that the interaction between MCM and TIMELESS was not essential for the function of TIMELESS in the replication checkpoint.

Next, we decided to check whether TIMELESS-M* could support full replication fork speed. EdU FACS showed that while TIMELESS depletion in clone O3 decreased the speed of DNA synthesis, expression of WT TIMELESS, but not the M* mutant, was able to restore the level of EdU incorporation (Fig. 5B). DNA fiber analysis confirmed that TIMELESS depletion decreased replication fork speed (Fig. 5C). While expression of WT TIMELESS fully restored replication fork speed, expression of TIMELESS-M* mimicked TIMELESS depletion, with significantly decreased replication fork speed (Fig. 5C). These data indicate that the interaction between MCM and TIMELESS is essential for supporting full replication fork speed in human cells.

Another function of TIMELESS and the FPC is in regulating the replication fork speed in response to oxidative stress: dissociation of TIMELESS from the replisome results in slower replication fork speed (7). We measured the levels of chromatin-associated TIMELESS and their changes after hydrogen peroxide treatment, using chromatin FACS (Fig. 5D**, S5**). Our data confirmed that oxidative damage led to significant decrease in chromatin-associated TIMELESS – endogenous and WT, while TIMELESS M* remained unaffected. These data support the model where leading edge TIMELESS regulates replication fork speed during normal replication and upon oxidative stress, and lagging strand TIMELESS mediates replication checkpoint signaling activation by facilitating the recruitment of TIPIN and CLASPIN to the chromatin.

## Discussion.

### Chromatin loading of the FPC during the initiation of DNA replication.

Synchronization experiments observing chromatin loading of the FPC components during S-phase entry showed that the FPC was present on chromatin before the loading of DNA polymerase alpha – the enzyme responsible for the initial DNA synthesis on both strands during replication initiation (Fig. 1A–D). Indeed, TIMELESS co-localized with MCM on G1-phase chromatin in our PLA assays, confirming that it was recruited to the origins before the start of DNA synthesis. However, inhibition of DNA synthesis by aphidicolin strongly decreased FPC chromatin loading (Fig. 1E–F), indicating that DNA synthesis is required for full FPC association with chromatin. This stepwise loading of the FPC is consistent with the presence of more than one FPC per replication fork. The leading edge FPC would load at the time of the CMG activation, as soon as the leading edge is established, and the ssDNA-associated FPC would load after DNA synthesis starts creating ssDNA spaces between Okazaki fragments. In agreement with this model, loading of both FPC instances would require CMG activation and kinase activities of CDK2 and CDC7/CDK1, while the second FPC would only be recruited once DNA synthesis starts and would therefore be blocked by aphidicolin.

Our data showed that TIMELESS depletion led to a decrease in chromatin binding of TIPIN and CLASPIN both during S-phase entry and during ATRi-induced replication origin firing (Fig. 2). We also confirmed the effect of TIMELESS depletion on CLASPIN chromatin binding in asynchronous cells using flow cytometry (Fig. 2D–E), in agreement with recently published data (13). TIPIN has been shown to recruit CLASPIN to ssDNA during replication checkpoint activation (4); however, the roles of TIMELESS/TIPIN in CLASPIN loading during the initiation of DNA replication have not been previously described. Our data demonstrate that TIPIN is unable to load onto chromatin in the absence of TIMELESS, clarifying that both TIMELESS and TIPIN are likely required for chromatin loading of CLASPIN. Interestingly, previous studies demonstrated that the loss of CLASPIN does not affect TIMELESS-TIPIN loading (5), supporting the model that the TIMELESS, and not CLASPIN, mediated the FPC chromatin association. Our study was also able to expand this observation to the second FPC instance, as TIMELESS-M* chromatin binding supported CLASPIN and TIPIN association with CSK chromatin in the absence of TIMELESS-MCM interaction (Fig. 4D). Our data support the model in which the role of TIMELESS in checkpoint regulation could be through recruiting CLASPIN to ssDNA/RPA.

### Position of TIMELESS at the replication fork

TIMELESS, as a component of the FPC, has been well described for its role in fork stabilization, interaction with DNA polymerases, as well as checkpoint activation (reviewed in(1)). These roles are not fully in agreement with its position at the leading edge of the replication fork, where it has been repeatedly identified by highly reliable structural studies (14–16). Additionally, TIPIN, TIMELESS’ obligatory binding partner, has been demonstrated to bind both dsDNA at the leading edge of the replication fork (14–16), and ssDNA/RPA(4), which is difficult to imagine simultaneously. Due to these inconsistencies, as well as our observation of a stepwise chromatin loading of TIMELESS during replication initiation, we used split-TurboID to shine more light on the position of TIMELESS at the replication fork. Surprisingly, the N-terminus of TIMELESS appeared to be in proximity to the C-terminus of AND-1, forming a functional biotin ligase that labeled replicative DNA polymerases, CDC45, CHK1, and other replication proteins (Fig. 3).

**Figure 6.**
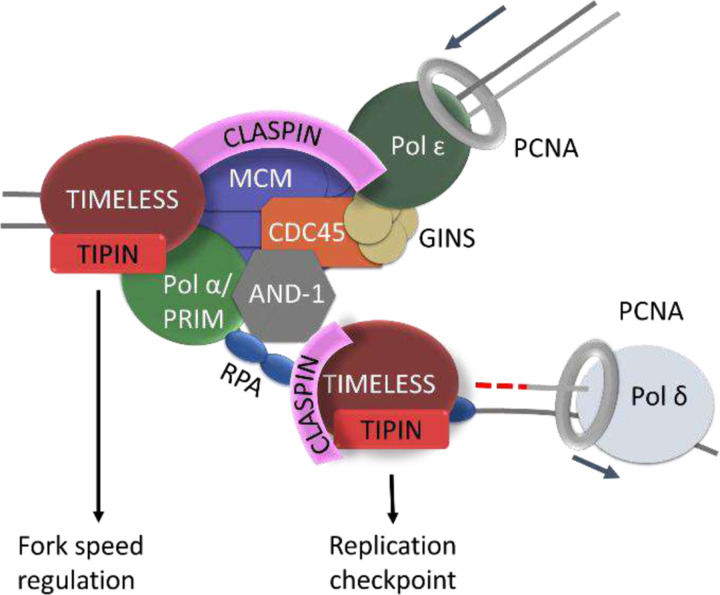
Proposed model illustrating two FPCs at two different positions at the replication fork.

AND-1 is sometimes regarded as one of the FPC components (36) despite there being no structural evidence of direct interactions between AND-1 and TIMELESS-TIPIN-CLASPIN. CLASPIN co-purified with AND-1 in co-immunoprecipitation experiments (37), which appeared to pull down the full replisome. A study in Xenopus egg extracts was able to co-purify AND-1 and TIPIN (38). Since both AND-1 and TIMELESS-TIPIN-CLASPIN interact with the CMG helicase, these co-purifications do not necessarily prove a direct interaction. One study indicated that AND-1 and CLASPIN both accumulate on ssDNA to promote S-phase checkpoint activation (39), which suggests a functional association, but on ssDNA and not at the leading edge of the fork where the FPC interacts with dsDNA (14–16). AND-1 could interact with ssDNA in its canonical position, but the reported accumulation of AND-1 on ssDNA (39) suggests that perhaps additional monomers or trimers of AND-1 are recruited to ssDNA during replication stress. Our successful combination of AND-1-TurboC/TurboN-TIMELESS in the absence of replication stress suggested that AND-1- and TIMELESS co-localized at unperturbed replisomes. While we cannot make a definitive conclusion, it is possible that the N-terminus of the leading-edge TIMELESS was in proximity to the long and flexible C-terminus of AND-1. However, POLD was biotinylated by the TIMELESS/TIMELESS combination, but not the TIMELESS/AND-1 combination, which indicates that the TIMELESS/TIMELESS contact was close to the lagging strand, while the TIMELESS/AND-1 and the TIMELESS/ORC6 contacts were not. We considered a possibility that two TIMELESS molecules coming together to produce this biotinylation could be from two different replication forks; however, if both tagged molecules of TIMELESS were located at the leading edges of their respective forks, biotinylation of DNA polymerase epsilon and CDC45, located on the directly opposite side of the CMG, is still very difficult to explain. Given that all three combinations used TIMELESS tagged at its N-terminus with the shorter (TurboN) fragment, this strongly supports our model where there are two instances of TIMELESS/TIPIN at the replication fork. We propose that there is an additional TIMELESS-TIPIN-CLASPIN complex at ssDNA/RPA on the lagging strand (Fig. 6), mediated by TIPIN-RPA interaction and supporting the background level of CHK1 phosphorylation by ATR in the absence of replication stress, known to be essential to suppress excessive origin firing (32).

In addition to checkpoint signaling, TIMELESS was shown to interact with PARP1 at the lagging strand (12, 40). TIMELESS interaction with PARP1 has been mapped (41) to amino acids 1029–1102, and the mutation that we introduced into the M* TIMELESS is not expected to affect this interaction. It is therefore reasonable to expect that PARP1-related functions of TIMELESS would also be CMG-independent, this should be experimentally explored in future studies.

### Significance of TIMELESS-MCM interaction.

Previous studies describing TIMELESS depletion phenotypes (5, 6, 42–45) documented the effect of depletion of both leading edge TIMELESS as well as lagging strand TIMELESS using either siRNA or a degron strategy, without separating the distinct roles of the two instances. Our system allowed us to specifically disrupt the leading edge TIMELESS without affecting the RPA-associated FPC. Our data indicated that leading edge TIMELESS is necessary for maintaining full speed of the replication forks, as well as efficient chromatin loading of TIPIN and CLASPIN. However, TIMELESS-M*, unable to interact with MCM, was sufficient to support the checkpoint function of TIMELESS, likely mediated by CLASPIN loading at ssDNA to support CHK1 phosphorylation by ATR (39). Disrupting TIPIN-RPA interaction was previously shown to block the checkpoint function of the FPC in biochemical experiments(4), and future studies will test whether this alteration could preserve the speed of the replication fork; our TIMELESS-centered system does not allow us to easily check that in cells. Nevertheless, we show here for the first time that the checkpoint function of the FPC does not require its interaction with the CMG and is mediated by a separate instance of TIMELESS. Our data demonstrate that the checkpoint function of the FPC is structurally and mechanistically separable from its role at the CMG. Given that TIMELESS is frequently overexpressed in cancers, providing resistance to chemotherapies and stimulating epithelial-to-mesenchymal transition as well as metastases (3, 46–48), our study offers a possible explanation for how the excess of TIMELESS protects cancer cells from DNA damage – without changing the number of active replication forks, additional FPC complexes at each fork could strengthen the checkpoint signaling, providing resistance to DNA damage, including chemotherapy.

## Materials and methods:

### Plasmids and cloning

For osTIR1 expression, we used pMK243-Tet-OsTIR1-PURO and AAVS1 CMV-OsTIR1(F74G) (a gift from Masato Kanemaki—Addgene plasmids # 72835 and #140536) plasmids (30, 49).

For mAID KI templates, homology arms were synthesized by Genscript in the pUC57 vector with the stop codon substituted with a BamHI site (5’ GGATCC 3’). The insert from plasmid pMK293 (mAID-mCherry2-Hygro) (Addgene plasmid # 72831 was a gift from Masato Kanemaki) (49), was cloned into the BamHI site of the synthesized plasmid, between the synthesized homology arms.

The TIMELESS C-terminus-targeting gRNA (ACGTGTCTATCTCTACCCCT) was expressed from the pSpCas9 BB-2A-Puro (PX459) v2.0 plasmid (Genscript).

TurboID fragments were cloned out of the 3xHA-TurboID-NLS_pCDNA3 plasmid that was a gift from Alice Ting (Addgene plasmid # 107171)(50) using the following primers TurboN_F: 5’-ATACGCGTTACCCCTATGACGTCCCAGA-3’; TurboN_R: 5’-ATGTTTAAACCAGAATCTGTTTAGCGTTCA-3’; TurboC_F: 5’-ATGATATCGGACAGCTGGACGGCGGGAG-3’; TurboC_R: 5’- ATGTTTAAACCTTTTCGGCAGACCGCAGAC-3’. TIMELESS ORF was cloned out of pcDNA4-Flag-Timeless, a gift from Aziz Sancar (Addgene plasmid # 22887)(51), using the following primers: Tim_Sgf1_F: 5’-ATGCGATCGCCATGGACTTGCACATGATGAA-3’ and Tim_Mlu1_R: 5’-ATACGCGTGTCATCCTCATCATCCTCAA-3’. AND-1 ORF was cloned out of the Origene plasmid #NM_007086 using SfaAI and MluI restriction sites. ORC6 ORF was cloned out of the Origene plasmid NM_014321 using SfaAI and MluI restriction sites.

For generation of the TIMELESS-M* mutant, the sequence corresponding to amino acids 276–302 was deleted from the TIMELESS gene, using Gibson Assembly-based cloning.

### Cell lines, cell culture, and transfections

U2OS (ATCC HTB-96) cells were grown in RPMI-1640 medium (Capricorn Scientific) supplemented with 10% FBS (GIBCO) and 1% penicillin-streptomycin (Invitrogen).

RPE-1 hTERT cells (ATCC CRL-4000) were grown in DMEM/F12 medium (GIBCO) supplemented with 10% FBS (GIBCO) and 1% penicillin-streptomycin (Invitrogen).

293T cells (ATCC CRL-3216) were grown in DMEM medium (GIBCO) supplemented with 10% FBS (GIBCO) and 1% penicillin-streptomycin (Invitrogen).

For generation of mAID2 TIMELESS-depleting cell lines by CRISPR (30, 49, 52), U2OS-based cells, expressing osTIR1 F74G mutant under the CMV promoter, were created by transfecting U2OS cells with plasmids expressing Cas9, gRNA targeting the C-terminus of TIMELESS gene, and a homologous template with the insert. The growth medium was changed 8 h after transfection, 2.5 μM DNAPK inhibitor was added for 48 h. KI cells were selected with hygromycin until the non-transfected control died, followed by single cell cloning and KI validation by PCR and western blot. Transfections were carried out using Lipofectamine 2000 (ThermoFisher), according to the manufacturer’s instructions. For validations of the knock-ins, genomic DNA was isolated using genomic DNA miniprep kit (Zymo Research, D3025). Primers used to validate the presence of the knock-in were as follows: TIMhtestF: 5’-CGACAATTGCTGGACAGCGAC-3’ and gtestR: 5’-GGATCCTTACTTGTACAGCTC-3’; the primers used to validate the absence of unedited TIMELESS allele were as follows: TIMhtestF and TIMhtestR: 5’-ATCTCCAGAGAGCTGCTGGGG-3’.

For TIMELESS WT or TIMELESS-M*-expressing cell line generation, mAID2 Clone O3 cells were transfected with the corresponding plasmid using Lipofectamine 2000 (ThermoFisher), according to manufacturer’s instructions, growth medium was changed 8 h after transfection, and cells were selected with G418 until the non-transfected control died, followed by single-cell cloning and a validation by western blot.

### Cell lysis, insoluble chromatin isolation, and western blots

Cells were lysed in TGN lysis buffer (50 mM Tris-HCl (pH 7.5), 150 mM NaCl, 50 mM NaF, 1% Tween-20, 0.5% Nonidet P-40, and protease inhibitors (Pierce #A32953)) for 20 min on ice. Lysates were cleared by centrifugation, and soluble protein was used for immunoprecipitation or mixed with 2X Laemmli Sample Buffer (Bio-Rad) and incubated for 7 min at 96 °C, followed by western blot. For nuclease-insoluble chromatin, pellets were suspended in NIB buffer (150 mM HEPES (pH 7.9), 1.5 mM MgCl_2_, 10% glycerol, 150 mM potassium acetate, and protease inhibitors) containing 1 μl of universal nuclease for cell lysis (ThermoFisher, 88700) per 100 μl of the buffer and incubated for 10 min at 37 °C on a shaker. Nuclease-insoluble chromatin was pelleted by centrifugation, washed with water, and resuspended in Laemmli Sample Buffer.

For western blot analyses, proteins were separated in 8%, 10%, or 12% SDS-polyacrylamide gels in running buffer (25 mM Tris, 192 mM glycine, 0.1% SDS), transferred onto PVDF membrane (BioRad, #1620177) in transfer buffer (25 mM Tris, 192 mM glycine, 10% ethanol), blocked with 5% non-fat milk (BioRad, #1706404) in TBST (TBS ThermoFisher, #BP2471, 0.1% Tween-20), incubated with an appropriate dilution of the primary antibody overnight at 4 °C, washed with TBST buffer, incubated with secondary antibody for 1 h at room temperature, washed with TBST, and developed using SignalFire^™^ Elite ECL Reagent (Cell Signaling, #12757P) and ImageQuant LAS 4000 imager (GE Healthcare) or X-ray film. Quantification of western blots was performed using Fiji/ImageJ (version 1.53u).

### Coimmunoprecipitation

293T cells were transfected with an empty vector or N-terminally FLAG-tagged wild-type TIMELESS and TIMELESS-M* expressing constructs, using Lipofectamine 2000 as per the manufacturer’s instructions. 48 h later, cells were lysed in TGN buffer as described above, and FLAG-tagged proteins were immunoprecipitated using anti-FLAG M2 affinity gel beads (Sigma-Aldrich), followed by elution with FLAG peptide (100 μg/ml in TGN buffer 2 h, 4°C).

### Split-Turbo ID experiments

293T cells were transfected using Lipofectamine2000, according to the instructions from the manufacturer. 48h after transfection, cells were incubated for 1h with 50 μM biotin, washed with PBS, and lysed in RIPA buffer (150 mM NaCl; 50 mM Tris-HCl pH 7.5; 1 % Triton X100; 0.1 % SDS, and protease inhibitors (Pierce #A32953)) on ice. After sonication using a Bioruptor (20 cycles, 30 seconds on; 30 seconds off), the lysates were cleared by centrifugation (14 000 g, 15 min) and incubated with streptavidin agarose (Thermofisher) for 16 h. The beads were then washed once with RIPA buffer, once with 1M NaCl, and two more times with RIPA buffer. Washed beads were then boiled with 1x Laemmli buffer (BioRad) and analyzed by western blot.

### Synchronizations and chromatin loading of replication proteins

In order to synchronize U2OS cells, 2 mM thymidine was added to ~25% confluent cells for 24 h. After thymidine removal, cells were washed once with warm PBS and allowed to recover in fresh medium for 5 h. Nocodazole was then added for 12 h to arrest the cells in G2/M. Dox (2 μg/ml) and 3-IAA (500 μM) for mAID1, or 5-pH-IAA (1.25 μM) for mAID2, were added at the same time as nocodazole. After release from nocodazole, cells were washed once with warm PBS, and incubated in pre-warmed medium with dox/aux or 5-Ph-IAA for the indicated periods of time. For the experiments with added inhibitors, cells were released from nocodazole, washed once with warm PBS, and incubated in pre-warmed medium with the indicated inhibitors for the indicated periods of time.

For RPE-hTERT synchronizations, cells were grown to confluency, growth medium was replaced with one without FBS and cells were incubated for 48 h to achieve G0 arrest. To release from G0, cells were passed 1:5 into complete medium. Cells entered S-phase between 15 and 18 h after release from G0.

In order to assess the loading of the various proteins on chromatin, samples were collected by trypsinization at the indicated time points, pellets washed once with ice-cold PBS and kept at −80 °C. To obtain the nuclease-insoluble fraction, thawed pellets were resuspended in CSK buffer (10 mM PIPES pH 7.0, 300 mM sucrose, 100 mM NaCl, 3 mM MgCl_2_, 0.5 % Triton X-100, and protease inhibitors (Pierce #A32953)), incubated for 5 min on ice, followed by a 5-min centrifugation (1000 × g, 4 °C). pellets were washed once more with CSK buffer, digested in CSK buffer with universal nuclease for cell lysis (ThermoFisher, #88700) for 10 min at 37 °C. Samples were mixed with 2x Laemmli Sample Buffer (BioRad) and boiled for 10 min before proceeding to western blot analysis. Quantifications were performed using ImageJ and GraphPad Prism 9.

### Proximity ligation assay (PLA)

In order to study the proximity of TIMELESS and MCM6 in S-phase and non-S-phase cells, cells were incubated with 10 μM EdU for 30 min, followed by a PBS wash, extracted with CSK buffer for 5 min on ice, washed twice with cold PBS, fixed with 4% paraformaldehyde for 10 min at room temperature, washed twice with PBS, and proceeded with the Proximity Ligation Kit according to the manufacturer’s instructions (Sigma Millipore).

### Antibodies

POLE1 (Santa Cruz, #sc-390785, 1:500), GAPDH (Santa Cruz, #sc-47724, 1:1000), pCHK1 (Cell Signaling, #2360S, 1:1000), MCM4 (Cell Signaling, #3228S, 1:300), CDC45 (Santa Cruz, #sc-55569, 1:500), GINS4 (Santa Cruz, #sc-398784, 1:300), H3 (Santa Cruz, #sc-517576, 1:1000), POLE2 (Santa Cruz, #sc-398582, 1:500), PCNA (Santa Cruz, #sc-56, 1:1000), FLAG (Sigma, F3165–1MG, 1:3000), polD1 (Santa Cruz, # sc-374025, 1:500), TIMELESS (Santa Cruz, #sc-393122), TIPIN (Santa Cruz, #sc-135580), CLASPIN (Santa Cruz, #sc-376773), H2AX (Santa Cruz, #sc-517336), MCM2 (Santa Cruz, #sc-373702), MCM7 (Santa Cruz, #sc-9966), RPA32 (Santa Cruz, #sc-56770), POLA1 (Santa Cruz, #sc-373884). For Chromatin FACS, CLASPIN (Santa Cruz, #sc-376773) antibody was used at 1:200 and TIMELESS antibody (Life Technologies, # MA534880) was used at 1:150 dilution. For PLA, TIMELESS antibody (Life Technologies, # MA534880) was used at 1:200 and MCM6 antibody (Novus Bio # H00004175-M04) was used at 1:1000.

### Inhibitors

ATRi AZD6738 (AstraZeneca), 5μM; DNAPKi NU7026 (Selleckchem), 2.5 μM; CDK1i Ro 3306 (Selleckchem), 5μM; CDK2i CVT-313 (Selleckchem), 5 μM; CDC7i XL413 (Selleckchem), 5μM; aphidicolin (MilliporeSigma), 2μM.

### Flow cytometry

For EdU FACS, cells were treated with 10 μM EdU for 30 min, trypsinized, washed with PBS, and fixed with cold 70% ethanol on ice for 30 min or at −20°С overnight. Cells were washed with PBS, and EdU staining was performed by using the EdU Click-iT kit (ThermoFisher, #C10632), according to the manufacturer’s instructions. For DNA staining 7-AAD (7-Aminoactinomycin D) (ThermoFisher, # A1310), FxCycle Far Red Stain (ThermoFisher, #F10348) or FxCycle^™^ PI/RNase Staining Solution (ThermoFisher, #F10797) were used. Samples were analyzed on FACSCalibur flow cytometer, and data were analyzed using FCSalyzer software. Alternatively, the samples were analyzed on Cytoflex 2L followed by data analyses using FlowJo. Software. GraphPad Prism 9 was used for statistical analyses.

### Chromatin FACS

For analyzing the presence of TIMELESS or CLASPIN on chromatin using flow cytometry, cells were collected by trypsinization, washed once with cold PBS and extracted with cold CSK buffer for 5 min on ice. Cells were then washed once with 1% BSA in PBS and fixed with 4% paraformaldehyde in PBS for 10 minutes at room temperature. Cells were washed once with 1% BSA and once with 0.5% BSA-0.25% NP40 in PBS. Cells were incubated with primary antibody dilutions in 0.5% BSA-0.25%NP40-PBS overnight at 4°C. Cells were then washed once with 0.5% BSA-0.25%NP40-PBS and incubated with secondary antibody dilution in 0.5% BSA-0.25%NP40-PBS for 1h at 37°C. After washed with 0.5% BSA-0.25%NP40-PBS and PBS-0.05% BSA, cells were stained with Far Red FxCycle (Thermofisher) according to manufacturer’s instructions. Stained cells were analyzed on Cytoflex2L (Beckman), with data processed using FlowJo and Graphpad Prism.

### DNA fiber analysis

Cells were pulsed with 20 μM CldU followed by 200 μM IdU to mark ongoing DNA synthesis. After trypsinization and PBS wash, cells were lysed by adding 6 μl of lysis buffer (200 mM Tris–HCl pH 7.4, 50 mM EDTA, 0.5% SDS) to 2ul of cell suspension and spread carefully with a plastic tip on a glass slide. After a 5 min incubation, the DNA was allowed to slide down the tilted slide. After drying the slides for 10 min at room temperature, the DNA was fixed by incubation in 3:1 methanol-acetic acid for 5 min, and dried for 8 minutes at room temperature. DNA was then rehydrated in PBS during 2 5-min incubations. DNA was then denatured in 2.5N HCl for 1h, followed by 2 5-min PBS washes. The samples were blocked in 5% BSA-0.1% Triton X-100 for 1h at 37°C, and incubated with primary antibodies (CldU #ab6326 1:50 and IdU BD#347580 1:66.6) at 4°C overnight. Slides were washed 4 times 5 min with PBS-0.1% Tween20 and incubated with secondary antibodies (1:150) for 1h at 37°C, washed 4 times 5 min with PBS-0.1% Tween20 and 2 times 5 min with PBS. The samples were then mounted with ProLong Diamond Antifade Mountant (ThermoFisher #P36961). The fibers were imaged with Nikon fluorescent microscope, and analyzed using ImageJ, at least 100 fibers per condition were measured.

## Supplementary Material

Supplementary Files

This is a list of supplementary files associated with this preprint. Click to download.

- Supplementaryfigureswithlegendsmay2026.pdf
